# Annual Meeting of the Brooklyn Dental Society

**Published:** 1878-03

**Authors:** 


### Proceedings of Societies.

### ANNUAL MEETING OF THE BROOKLYN DENTAL SOCIETY.

The Tenth Aniversary of this body was held in Brooklyn, on the afternoon and evening of December 14th. The afternoon session was held at the office of Dr. H. G. Mirick, No. 156 Clinton street, when some discussion was had upon some subjects of interest to the profession, and the more recent discoveries that have been made in connection with dentistry. No business was transacted and the proceedings took the form of a scientific discussion.

The meeting was well attended, nearly all members of the society being present. At present the organization numbers thirty-six members, with a board of officers and committees composed as follows:

President, O. E. Hill; Vice-President, A. H. Brockway; Recording Secretary, C. P. Crandall; Corresponding Secretary, W. H. Atkinson; Treasurer, F. W. Dolbeare; Librarian, C. E. Mensch.

COMMITTEES.

Executive—A. N. Chapman, Wm. Fishbough, W. A. Campbell.

Membership—Wm. Jarvis, J. H. Race, C. W. Harreys.

Ethics—G. A. Mills, C. D. Cooke, C. E. Mensch.

Subjects—C, P. Crandall, A. H. Brockway, O. E. Houghton.

Clinics—H. G. Mirick, W. H. Atkinson, G. L. Mason, C. H. Biddle, J. N. Farrer.

Monthly Meetings are held at the houses of the members where papers are read and discussed, and matters of professional import discussed. The code of ethics adopted by the society is very rigid, and the requirements for membership exacting. The society will be actively engaged during the winter in getting a bill through the Legislature regulating the requirements which must be satisfied before any one can be allowed to practice dentistry in this State.

Everett Hall, No, 396 Fulton street, was the place selected for the dinner, and seven o'clock was named as the hour.

Among those who were present were Dr. O. E. Hill the president; Dr. S. G. Armor, of the Long Island College Hospital; Professer Garrettson and Professor McQuillen; Dr. S. S. White, of Philadelphia; Dr. C. F. Wheeler, Albany, N. Y.; Drs. John B. Rich, William A. Bronson, E. A. Bogue, S. G. Perry, W. St. George Elliot, W. H. Atkinson, C. E. Francis, J. G. Ambler, C. A. Woodward, William Carr, J. W. Clowes, N. W. Kingsley and Benjamin Lord, from New York.

From Brooklyn there were Drs. William Jarvie, Jr., H. G. Mirick, G. A. Mills, D. S. Skinner, A. N. Chapman, John Scott, O. E. Houghton, J. H. Race, C. A. Marvin, M. E. Elmendorf, A. H. Brockway, Charles H. Biddle, W. H. Fishbough, T. Frye, E. H. Stelle, A. K. Johnstone, F. W. Dolbeare, M. L. Thompson, J. C. Monroe, C. P. Crandall, C. D. Cooke and many others.

The guests sat down promptly on time at 7 o'clock, and the ensuing two hours were devoted to the joys of the table and the pleasures of social intercourse.

THE TOASTS AND SPEECHES

The President, calling the assembly to order, welcomed the members of the Society and the invited guests, and expressed the pleasure which he felt in seeing them together and in meeting so many professional brethren. He said that in the ten years of the life of the Society there had not occurred a single event to mar the cordial friendship that existed among the members. They had been prosperous as a Society, and it was eminently fitting that they should celebrate the tenth anniversary of their organization. He congratulated the members on the good fellowship, kind feeling and strong professional bands that had held them together so long, and expressed the hope that the future would see the bonds of union strengthened and the usefulness of the Society widely increased. He urged the members to renewed and noble aims in the profession which they had chosen.

Dr. Hill then called upon Dr. H. G. Mirick to read a history of the Society.

Dr. Mirick responded. The following is an abstract of

THE HISTORY.

The Society was first organized upon the suggestion of Dr. George A. Mills, who invited a number of gentlemen to meet together to discuss the feasibility of the scheme. Thirteen dentists accepted the invitation. Their names were, O. E. Hill, E. L. Childs, J. C. Monroe. John Scott, Thomas Frye, A. H. Brockway, L. E. Brockway, Wm. Jarvie, Jr., N. M. Abbott, H. E. Bird, George E. Betz, George A. Mills and H. G. Mirick. A permanent organization was effected, with H. G. Mirick, as President; C. D. Cooke, Vice-President; E. L. Childs, Recording Secretary; Wm. Jarvie, Corresponding Secretary; and S. C. Moore, Treasurer; George A. Mills, O. E. Hill and John Scott constituted the First Executive Commitee. The original name of the Society, was the Brooklyn Society of Dental Science and Art, but that was afterward changed to Brooklyn Dental Society. In 1869 the Society was incorporated. The Society now numbers thirty-six members, and is placed on a prosperous and flourishing basis. A dental infirmary was started in the earlier years of the Society, but was discontinued. There have been three deaths of active members, and the position of President has been held by nine different gentlemen.

Dr. Mirick spoke further about the past history of the Society, and closed amid loud applause.

The first regular toast was "The Science of Dentistry," which was responded to by Dr. W. H. Atkinson, of New York, who spoke upon the claims of science, and said that dentistry took hold on the three kingdoms of the scientific world. In the enamel of the teeth they had the mineral kingdom of crystallization. In the dentine, the vegetable or cellular formation, and in the pulp the protoplasmic form of nature. To be good dentists they must be masters of the factors of function in the human body.

The second toast was "Dental Surgery," which was responded to by Professor Garrettson, M. D., of Philadelphia, who spoke at length upon the relations of dental surgery to medicine. He said that medicine was the great alma mater of the specialties that had shot off from the main source for years, and they must go back to it to find the source and the spring of them all. He claimed that denistry was not a profession but one of the specialties of the great science of medicine.

The next speaker was.

DR. S. G. ARMOR, of the Long Island College Hospital, who responded to the toast, "The Science of Medicine." In the course of his remarks, and after alluding to the high calling of the medical profession, Dr. Armor, said: "But the physician, has, beside, to contemplate the body under disease, and to bring to his aid all the resources of nature to counteract and control that which disturbs its well being. But the body is not the only object of its care. We are callled on to treat the entire man physical, moral and intellectual. Who is sufficient for these things? What more can I say of the greatness of our art, a science and an art whose aim is the promotion of human health, and the relief of human suffering?

"And this science and art have direct and intimate relations to dental education. Medicine should be a unit in all its departments. Dental surgery is a specialty of medicine, just as gynæcology, ophthalmology and all other special departments of study are specialties. We claim you as part of the general family, and we sympathise and rejoice with you in every effort to advance dental education. We are proud of the position you are taking—proud of your colleges— and especially proud of your reputation. In the department of Dental Surgery, America leads the world. I wish I could say as much of what you have called "The Parent Science." But we are working bravely, striving for better preliminary education of our students, grading their studies, lengthening their college terms, giving better opportunities for clinical study and in all this effort to advance the interest and usefulness o noble profession. We know that we have your sympathy and co-operation.

"Common lines of thought, common studies, common sympathies. We have not a subject of interest in general medical literature that does not interest the student of dental surgery. The lines diverge, and yet touch at every page of our literature. We can not discuss a question of physiology or general pathology that does not interest the student of oral surgery; for there is not a tissue, a growth, or isolated cell of the human body that is not affected by its general condition. Dental surgery, then, should grow out of a broad and liberal culture in the several departments of a general medical education, especially in the departments of anatomy, physiology, principles of surgery, pathology, chemistry, and general therapeutics. In the cultivation of these branches let us strive with each other, and like the glorious youth of Longfellow, let us not be content to travel in the low grounds and valleys of professional life, but take to the higher plains and mountains, determined, even amid warnings and discouragements, to reach the dizzy height and inscribe upon our banner 'Excelsior.'

"Thanking you, gentlemen, for the honor you have conferred upon me in inviting me to your banquet, and for asking me to respond to the toast complimentary to a noble profession in whose practicing and teaching I have devoted the best years of my life, I close with the earnest hope that dental science and art may ever continue to occupy the proud and honorable position it does in the oral surgery of the world."

The next toast was: "Ten Years of Progress," which was responded to by Professor McQuillen, of Philadelphia, who alluded to the progress which had been made in science during the last decade, particularly in its relations to dental surgery. He urged the necessity of a broader education in the training of dental surgeons, and incidentally, advocated a compulsory system of education by the state at large.

The toast of "Sister Organizations" was responded to briefly by Dr. Lord, of New York, and Dr. Stalton, of New Jersey.

Dr. Dolbeare, of Brooklyn, then read a brief poem, descriptive of the progress of the Society during the past ten years, which was well received, and the exercises closed with the response to the toast. "A Sound Mind in a Sound Body," by Dr. Rich, in which he advocated the necessity of health for the proper pursuit of the profession.

By this time it was nearly midnight, and the assemblage dispersed, after a thoroughly pleasant and enjoyable evening, in which there had not been a single element to mar the perfect success of the affair.

